# Brain Oscillatory Correlates of Altered Executive Functioning in Positive and Negative Symptomatic Schizophrenia Patients and Healthy Controls

**DOI:** 10.3389/fpsyg.2016.00705

**Published:** 2016-05-10

**Authors:** Barbara Berger, Tamas Minarik, Birgit Griesmayr, Renate Stelzig-Schoeler, Wolfgang Aichhorn, Paul Sauseng

**Affiliations:** ^1^Department of Psychology, Biological Psychology, Ludwig-Maximilians UniversityMunich, Germany; ^2^Department of Psychology, University of SalzburgSalzburg, Austria; ^3^University Clinic for Psychiatry and Psychotherapy, Christian-Doppler-Clinic, Paracelsus-Medical Private UniversitySalzburg, Austria

**Keywords:** positive and negative symptomatic schizophrenia, brain oscillations, executive functions, working memory, fronto-parietal network, theta, gamma

## Abstract

Working Memory and executive functioning deficits are core characteristics of patients suffering from schizophrenia. Electrophysiological research indicates that altered patterns of neural oscillatory mechanisms underpinning executive functioning are associated with the psychiatric disorder. Such brain oscillatory changes have been found in local amplitude differences at gamma and theta frequencies in task-specific cortical areas. Moreover, interregional interactions are also disrupted as signified by decreased phase coherence of fronto-posterior theta activity in schizophrenia patients. However, schizophrenia is not a one-dimensional psychiatric disorder but has various forms and expressions. A common distinction is between positive and negative symptomatology but most patients have both negative and positive symptoms to some extent. Here, we examined three groups—healthy controls, predominantly negative, and predominantly positive symptomatic schizophrenia patients—when performing a working memory task with increasing cognitive demand and increasing need for executive control. We analyzed brain oscillatory activity in the three groups separately and investigated how predominant symptomatology might explain differences in brain oscillatory patterns. Our results indicate that differences in task specific fronto-posterior network activity (i.e., executive control network) expressed by interregional phase synchronization are able to account for working memory dysfunctions between groups. Local changes in the theta and gamma frequency range also show differences between patients and healthy controls, and more importantly, between the two patient groups. We conclude that differences in oscillatory brain activation patterns related to executive processing can be an indicator for positive and negative symptomatology in schizophrenia. Furthermore, changes in cognitive and especially executive functioning in patients are expressed by alterations in a task-specific fronto-posterior connectivity even in the absence of behavioral impairment.

## Introduction

Schizophrenia is a complex disorder comprised of widespread affective, cognitive, and behavioral disturbances and disruptions. The most widely used sub-classification of schizophrenia is the division into positive (hallucinations and delusions or confused thoughts and speech) and negative (anhedonia and withdrawal) symptomatology. Further core characteristics of the disorder are cognitive deficits such as disrupted memory processes, executive functioning, and attention. Such cognitive disturbances are usually equally present in patients showing predominantly negative or positive symptoms.

At the cortical level, patients suffering from schizophrenia (SZ) show large scale structural changes and alterations in the functional architecture of the brain. The condition is associated with significant gray and white matter volume losses (Kiriakopoulos et al., 2008; Huhlshoff Pol et al., 2002; Roalf et al., 2015; van Erp et al., 2015). Furthermore, fMRI studies indicate altered hemodynamic responses in distributed brain regions among SZ patients from sensory areas (Haenschel et al., 2009) to regions associated with higher-cognitive functioning (for a review see Ettinger et al., 2015). Electrophysiological studies also revealed dysfunctional electrophysiological activity in various parts of the cortex (Haenschel and Linden, 2011; Uhlhaas and Singer, 2015). The widespread structural and haemodynamic changes are a hallmark of schizophrenia. Yet the PFC seems to be disproportionately affected regarding structural but also functional changes (Wen et al., 2011; Zhou et al., 2015), and appears to contribute to patients' cognitive impairment (WM loss, attention deficit, impaired executive functioning, etc.).

Indeed several studies confirmed that dysactivation in the PFC is correlated with WM and executive functioning deficits (see Teffer and Semendeferi, 2012 for reviews; Etkin et al., 2013). In patients suffering from schizophrenia hypofrontality is most often reported where SZ patients display a pronounced decrease in prefrontal activation compared to healthy control participants while performance is usually but not necessarily affected (e.g., Carter et al., 1997; Callicott et al., 2003). Furthermore, Kerns et al. (2005) show that patients display reduced activity in the anterior cingulate cortex (ACC) during cognitive tasks, and that this reduction is associated with impaired conflict monitoring. In a meta-analysis by Glahn et al. (2005) clear support for hypofrontality in patients as compared to healthy controls was found. But the opposite seems to hold true in many cases; where patients consistently show increased activation (hyperfrontality) in prefrontal regions during working memory operations. Such hyperfrontality is normally interpreted as a compensatory mechanism (Callicott et al., 2003; Manoach, 2003). For example, Karlsgodt et al. (2007) found a strong positive association between the amount of prefrontal activation and performance. Manoach et al. (2000), on the other hand, report an increased prefrontal activation in SZ patients while performance was significantly impaired as compared to healthy controls. Taken together these studies indicate a less than uniform picture about frontal activation in patients with SZ during working memory task performance. What becomes clear, however, is that aberrant frontal activation during cognitive task performance and executive control is prominent in patients and might reflect general dysfunctions due to the disorder as well as mechanisms to compensate for those.

Thus, investigating WM, attention, executive control, and altered activity in the PFC seem to be a key factor for understanding the cognitive deficits associated with SZ. However, the above mentioned studies do not tell the entire story. Converging evidence suggests not only overall activation/deactivation as measured with brain imaging techniques but also transient and sustained EEG oscillatory mechanisms are crucial for understanding brain functioning in healthy as well as patient populations (Voytek and Knight, 2015). The exact timing of neuronal activity is important for information processing (e.g., O'Keefe and Recce, 1993; Singer and Gray, 1995) and rhythmic activity ranging from below 1 Hz to well above 100 Hz provide a time frame for neuronal synchrony (e.g., Buzsaki and Wang, 2012). Slow oscillations (e.g., theta 4–7 Hz) are associated with co-ordinating processes that span larger networks sometimes distributed across several cortical areas. Frontal theta oscillations—consistently found to originate in the ACC (e.g., Tsujimoto et al., 2006) are sensitive to task difficulty and prominent during maintenance and manipulation of information, during sustained attention and during unspecific processes of cognitive resource allocation (e.g., Gevins et al., 1997; Sauseng et al., 2007, 2010; Mitchell et al., 2008; Berger et al., 2014; Cavanagh and Frank, 2014). By contrast, gamma oscillations are mainly reported in connection to local information processing and co-ordination of activity in rather small networks. Gamma frequency oscillations are positively correlated with the BOLD signal and the firing of local neuronal assemblies (e.g., Roux and Uhlhaas, 2014).

A series of studies emphasizes that WM and attention deficits in patients with schizophrenia are accompanied by alterations in brain oscillatory activity. Gamma band-specific changes in the PFC are some of the most often reported oscillatory correlates of SZ, usually exhibiting lowered resting-state and task specific gamma activity (e.g., Cho et al., 2006; Basar-Eroglu et al., 2007; Gandal et al., 2012; Senkowski and Gallinat, 2015). However, contradicting findings also exist where SZ patients displayed stronger PFC gamma activity both during resting and cognitive operations in comparison to healthy control subjects (see e.g., Moran and Hong, 2011 or Senkowski and Gallinat, 2015 for a review). The dysregulation of GABAergic inhibitory neurotransmission—and hence imbalance between inhibition and excitation—is thought to underlie prefrontal gamma band changes. It might be one potential key mechanism responsible for many of the cognitive deficits in schizophrenia (Dasklakis et al., 2007; Uhlhaas and Singer, 2010; Lisman, 2012).

Another brain oscillatory pattern commonly found in SZ is increased low frequency brain activity in particular at theta, but also at delta frequencies (see Lisman, 2012 for a review). However, findings are also reported in the opposite direction where patients suffering from SZ display significantly less theta power than healthy control subjects (e.g., Koychev et al., 2012). This frontal—and especially medial frontal—slow frequency aberration in patients with SZ is well in line with their experienced deficits in WM and attention processes and might either portray the impairment itself or alternatively, a compensatory mechanism to employ prefrontal resources to counter the deficit in cognitive processing. The controversial results regarding theta increase or decrease, however, are somewhat difficult to explain as the neurobiological mechanisms behind low frequency changes in SZ are less well identified than the ones behind fast frequencies. Furthermore, it is unclear whether low and high frequency changes are related or whether they are manifestations of two entirely separate neurobiological mechanisms (Uhlhaas and Singer, 2014).

Recent research identified local oscillatory and interregional coupling mechanisms driving allocation of attention and WM operations (Sauseng et al., 2005, 2010; Mizuhara and Yamagutchi, 2007; Wang et al., 2009; Ishii et al., 2014). Thereby, fronto-parietal neural synchronization seems to play a major role for executive functions. Given that executive control and WM operations are described as deficient in patients with SZ, it would make sense if the underlying neural correlates were affected by the disorder as well. And indeed, as Griesmayr et al. (2014) could show theta coherence between frontal and posterior cortical brain areas seems to be strongly decreased during a WM task in SZ patients as compared to healthy controls. In line with this, in a PET study, Meyer-Lindenberg et al. (2001) found aberrant fronto-posterior connectivity patterns as well as hypofrontality located in the ACC during a WM task in patients suffering from schizophrenia as compared to control subjects. Additionally, Popov et al. (2015) found decreased fronto-parietal connectivity (indexed by ACC theta phase to inferior parietal gamma amplitude coupling) in patients during increased demand for executive control. These findings are well in line with work demonstrating a general dysconnectivity in schizophrenia (see Uhlhaas and Singer, 2010; Schmitt et al., 2011). Rogasch et al. (2014), for instance, present evidence for dysconnectivity between task-dependently interacting cortical and subcortical areas in SZ as compared to healthy controls. Furthermore, they conclude that the inability to generate gamma oscillations in the PFC seems to be linked to higher positive symptomatology and cognitive impairment.

Findings highlighting specific symptoms being differentially linked to altered functional brain activity indicate that patients suffering from predominantly positive or predominantly negative symptoms might have differentially affected electrophysiological correlates of cognitive and especially executive functioning. This might also explain some of the above discussed controversial fMRI/EEG results. Lisman and Buzsaki (2008) for example argue that dysregulations of theta and gamma oscillations could result in very different symptoms; the former is responsible for confusion in the order of thoughts or percepts while the latter for memory deficits. Herrmann and Demiralp (2005) found that negative symptoms correlate with a decrease in gamma activity, whereas increased gamma activity was linked to positive symptoms such as hallucinations. Despite their high value for understanding the disorder itself, it might be a critical issue that most of the electrophysiological studies differentiating between positive and negative symptomatology look at the resting state activity in patients and healthy controls only. Importantly, however, in order to link findings regarding neural correlates of executive control and working memory deficits in SZ patients with their dysfunctional neuronal processes, it is vital to investigate patients during the execution of such a task. More specifically, we wanted to investigate and compare patients with predominantly positive and predominantly negative symptomatology regarding central executive difficulties in working memory. Additionally, the aim of this study was to investigate potential brain oscillatory signatures of central executive dysfunctioning at fast as well as slow EEG frequencies.

For this purpose we examined the difference between patients with predominantly negative symptoms, patients with predominantly positive symptoms and healthy control subjects that were tightly matched in gender, age and highest level of education during executing a visuo-spatial delayed-match-to-sample working memory task with high executive function demand while EEG was recorded. We investigated whether the two patient groups and healthy controls display differences in neuronal processing in the theta and gamma frequency bands in the ACC as well as the posterior parietal cortex (right and left BA40). As outlined above these frequency bands seem to be critically involved in complex working memory operations and the specified regions of interest are crucial for fronto-parietal executive control processes. Finally, we wanted to know whether patients and control subjects differ in terms of fronto-parietal communication during a working memory task with high central executive load. Our study aims at understanding alterations of working memory related brain oscillatory activity in patients suffering from schizophrenia predominated by different symptomatologies (negative and positive). Thereby we are trying to fill a gap in the understanding of this complex disorder as, to our knowledge, there is no research that has investigated these schizophrenia sub-populations in such a manner.

## Materials and methods

The here reported data are a reanalysis of part of the data previously published by Griesmayr et al. (2014). In comparison to this earlier publication, however, the analyzed sample was divided according to symptomatology; i.e., patients suffering from predominantly positive symptomatic and predominantly negative symptomatic schizophrenia. Moreover, in the current paper data were analyzed in EEG source space in order to identify the effects in specific regions crucially implicated in the fronto-parietal control network.

### Participants

Twenty-seven patients meeting ICD 10- criteria for schizophrenia participated in the study. Patients were recruited from the in- and outpatient facilities of the “Christian-Doppler Klinik Salzburg.” They had normal or corrected to normal vision and intact color vision. None of the patients showed a history of neurological illness or had alcohol or substance abuse within the last month prior to study participation. Further criteria for participation were that pharmacological treatment was stable and that they did not have acute florid symptomatology. Seven participants had to be excluded from data analysis because they could not finish the task (*n* = 3), the EEG was too heavily contaminated with artifacts (*n* = 4). Severity of clinical symptomatology was assessed with the PANSS (Positive and Negative Syndrome Scale; Kay et al., 1987) by trained psychiatrists. The obtained scores were used to classify patients either as “predominantly negative symptomatic” or “predominantly positive symptomatic” (later referred to as NEG and POS, respectively) which resulted in the two patient sub-groups (NEG and POS) with 10 patients each (see Table 1 for details and statistical comparisons). The two patient groups were matched according to age, gender, and highest education level. The diagnostic subgroups of the NEG and POS groups were paranoid schizophrenia (NEG: *n* = 7; POS: *n* = 9), undifferentiated schizophrenia (NEG: *n* = 1) and schizoaffective disorder (NEG: *n* = 1; POS: *n* = 2). Patients received treatment with atypical (NEG: *n* = 9; POS: *n* = 5), typical (POS: *n* = 2), and combined atypical and typical (NEG: *n* = 1; POS: *n* = 3) antipsychotics. Additionally, some participants received low doses of benzodiazepines (POS: *n* = 3) and/or antidepressants (NEG: *n* = 5; POS: *n* = 3). The mean chlorpromazine equivalent for NEG was 321.67 mg/day (SD = 257.92) and for POS 806.68 mg/day (SD = 326.87) (see Möller et al., 2000; Woods, 2003; see Table 1 for statistical comparisons).

**Table 1:** **Demographic and clinical characteristics (*p*-values represent the significance of the performed Kruskal–Wallis tests between groups, except gender, and education, which were tested with chi-square tests)**.

	**Controls**	**Negative symptomatic sub-group**	**Positive symptomatic sub-group**	***p*-values**
N	10	10	10	
Age	33.50 ± 2.87	31.60 ± 2.59	32.80 ± 2.43	*p* = 0.925
Gender (male/female)	6/10	7/10	8/10	*p* = 0.621
PANSS overall	–	78.66 ± 23.91	77.11 ± 20.91	*p* = 0.880
PANSS neg.sympt.	–	24.67 ± 3.03	14.56 ± 1.51	*p* = 0.015
PANSS pos.sympt.	–	16.67 ± 2.12	21.67 ± 2.78	*p* = 0.138
Education				*p* = 0.312
*Higher degree*	3	3	0	
*A-levels*	5	4	3	
*Apprenticeship/professional school*	1	1	3	
*Elementary school*	1	2	4	
Colrpromazine (mg/day)	–	321.67 ± 257.92	806.68 ± 326.87	*p* = 0.002

The 10 healthy control participants (later referred to as CON group) are a sub-set of the 21 healthy controls used by Griesmayr et al. (2014). They were matched to the two patient groups according to age, gender and level of highest education. Prior to testing they completed the Brief Symptom Inventory (Franke, 2000) which screens for clinically relevant psychological stress symptoms and the Structured Clinical Interview (SCI-I) to check for any mental disorders. Moreover, participants were excluded if they had any neurological diseases or first-degree relatives with a psychiatric disorder as well as when they reported alcohol or substance abuse within the last month prior to study participation.

All participants gave a written informed consent and were monetarily reimbursed for participation. The study was approved by the ethics committee of the Federal State of Salzburg and conducted in agreement with the Declaration of Helsinki.

### Experimental procedure

**Figure 1:**
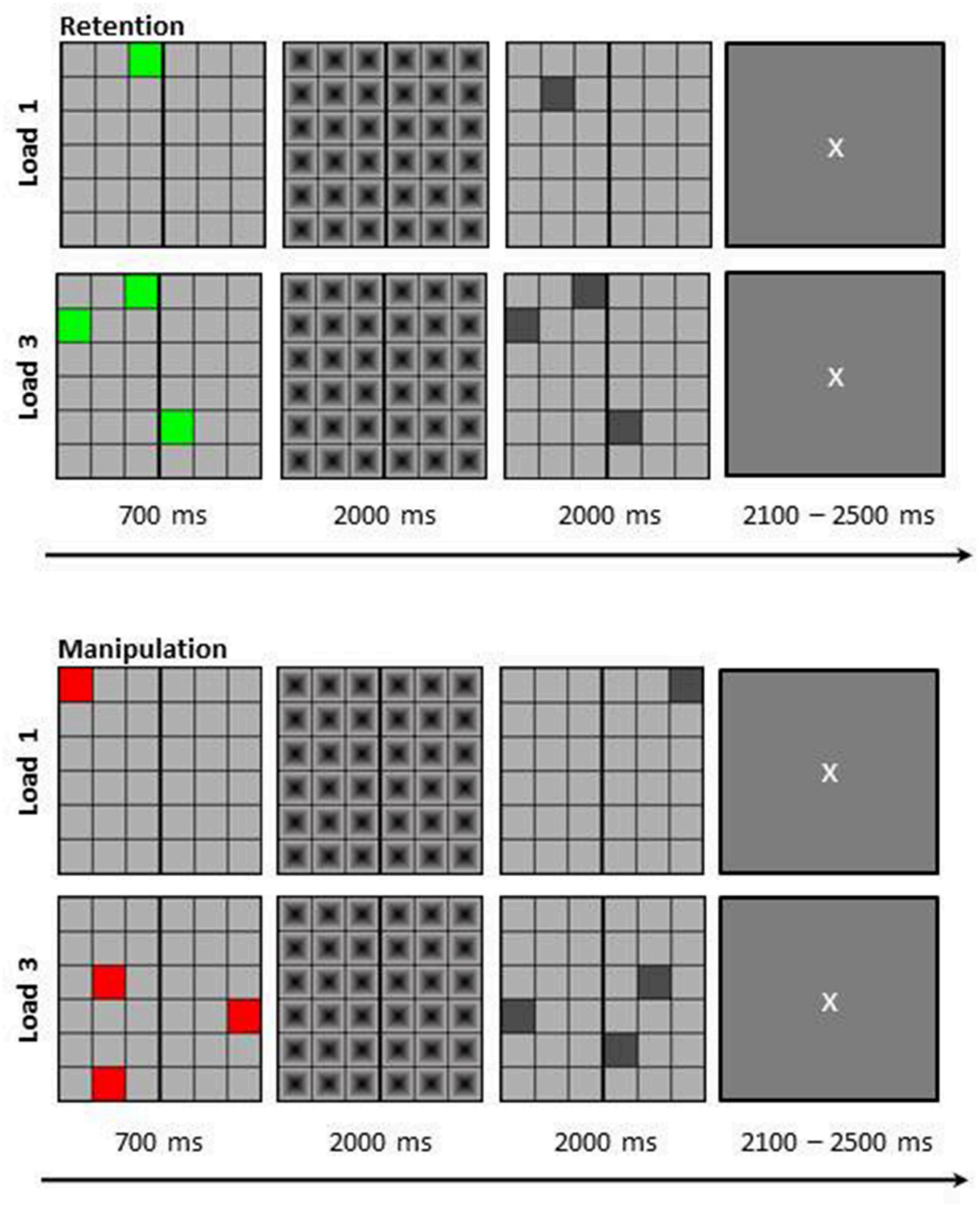
**Schematic depiction of the visuo-spatial delayed match to sample working memory task**. Participants had to either retain the positions of the colored squares (Retention) or mirror their positions around the vertical line (Manipulation). The load was either one item (L1) or three items (L3). Figure taken with permission from Griesmayr et al. (2014).

Participants performed a visuo-spatial delayed-match-to-sample working memory task in a dimly lit room. The stimuli were presented centrally on a computer screen with Presentation® 0.71. The memory set was presented at the beginning of each trial for 700 ms as a 6 × 6 matrix (visual angle of 9.2° × 9.2°) in which either one (load 1) or three (load 3) colored squares were presented. Additionally, if the squares were green, participants simply needed to retain their position for a delay period of 2000 ms (retention task); while if the squares were red they needed to mentally rotate their positions around a vertical line in the middle of the matrix (manipulation task) during the delay interval. During the 2000 ms delay period a mask was presented to prevent afterimages. Thereafter, a probe matrix was presented in which one or three (depending on load) gray squares were presented for a further 2000 ms. Participants then needed to indicate by mouse button press whether the square locations in the probe matrix matched the memory set or not (or the mentally rotated version thereof, depending on task). For non-match trials one of the squares in the probe matrix was shifted for one location. Between trials participants saw a fixation cross (duration jittered between 2100 and 2500 ms; see Figure 1 for depiction of trial structure and stimulus example). Participants were instructed to answer as correctly as possible.

In total 224 trials were presented which resulted in 56 trials per condition—retention load 1 (RETL1), retention load 3 (RETL3), manipulation load 1 (MANL1), and manipulation load 3 (MANL3)—of which half were match and half were non-match. Trials were presented in randomized order and a practice block was carried out before the start of the actual experiment.

### EEG recording

Data were recorded from 28 Ag-AgCl ring electrodes (Easycap®) mounted according to the international 10–20 system: Fp1, Fp2, F7, F3, Fz, F4, F8, FC3, FCz, FC4, T3, C3, Cz, C4, T4, CP5, CPz, CP6, T5, P3, Pz, P4, T6, PO3, PO4, O1, Oz, O2; against two reference electrodes placed on the earlobes. The ground electrode was placed on the forehead and eye movements were recorded with an electrode each above and next to the right eye. The EEG signal was registered between 0.016 and 80 Hz with a sampling rate of 1000 Hz and a notch-filter set at 50 Hz using a BrainAmp MR+ amplifier (Brain Products®). Impedances were kept below 15 kΩ.

### Data analysis

#### Behavioral data analysis

For behavioral data analysis IBM SPSS Statistics 22 was used. The accuracy (percentage of correctly performed trials) was analyzed using Wilcoxon Signed Rank test to examine the difference in performance comparing low-load and high-load conditions, as well as, the retention and the manipulation task, irrespective of the group of participants. The impact of the participants' group on the accuracy was further tested with a series of Kruskal-Wallis tests.

#### EEG data analysis pre-processing

For EEG data analysis Brain Vision Analyzer 2.0 (Brain Products®) and in-house Matlab R2014b scripts (Math Works®) were used and statistical analyses were performed with IBM SPSS Statistics 22 and Matlab. Data were first offline re-referenced to digitally linked earlobes and high-pass filtered with a low cut-off at 1 Hz (48 db/Oct Butterworth Zero Phase IIR Filter as implemented in BrainVision Analyzer 2.0). In order to remove horizontal and vertical eye movements and blinks, independent component analysis (ICA) ocular correction was applied to the filtered raw EEG data. Remaining artifacts were removed by visual inspection. Then data were segmented (from 1000 ms pre-stimulus onset to 3000 after stimulus onset, in respect to the memory set) for every condition separately as the focus of the analysis was on the delay interval; i.e., when participant retained the location of the square(s) or mentally rotated them. On average this resulted in 45.97 (SD = 4.82) artifact free trials for RETL1, 46.7 (SD = 5.02) for RETL3, 44.4 (SD = 4.77) for MANL1 and 47.57 (SD = 4.13) for MANL3. An estimation of the electrophysiological activity at source space was then calculated using the LORETA algorithm implemented in Brain Vision Analyzer 2.0 (see Pascual-Marqui et al., 1994). The algorithm transforms the scalp-level EEG data into a time series of current source density in pre-defined regions of interest (ROIs) in 3D-source space for each single trial. The ROIs were specified in line with the fronto-parietal network hypothesis outlined in the introduction; this resulted in one ROI in the bilateral ACC and one in the right BA40 (posterior parietal cortex bordering the IPS) and one in the left BA40.

#### Instantaneous amplitude

To obtain the instantaneous amplitude of theta and gamma frequencies in these regions, two individual continuous complex Morlet wavelet transformations were calculated for slow and fast frequencies, respectively. For slow frequencies a 5-cycle Morlet wavelet was used to analyse the frequency range from 3 to 15 Hz in 13 frequency steps. For fast frequencies a 10-cycle Morlet wavelet transform was calculated from 30 to 80 Hz in 6 frequency steps. Any further analysis was done using in-house Matlab R2014b scripts (Math Works®). In order to account for outliers we used a conventional cut-off of ±3.29 standard deviations for every subject and every data point across trials. Values that exceeded this individual cut-off were set to the value representing ±3.29 times the standard deviation. This was done to attenuate the impact of extreme values without having to remove the whole trial. For the calculation of changes in the instantaneous amplitude of theta and gamma oscillations during the 2000 ms retention/manipulation interval the instantaneous amplitude values were first averaged across trials. The power values of both, theta and gamma frequencies were decibel transformed (see (Cohen and Ridderinkhof, 2013) for more details) according to a baseline period from 500 to 200 ms prior to stimulus onset. Decibel conversion was used in order to ensure comparability between all frequencies, conditions, groups or subjects and time points as it brings all data to the same scale. Frequency bins between 4 and 8 Hz were averaged to obtain a theta band, and frequency bins between 30 and 50 Hz and between 50 and 80 Hz were averaged into a slow and a fast gamma band, respectively. Finally, the decibel transformed amplitude values for the three frequency bands were averaged into four time windows of 500 ms each between stimulus-offset and probe-onset. For statistical comparisons the average decibel transformed amplitude values for each time window were exported for each condition (RETL1, RETL3, MANL1, and MANL3) and each individual subject and analyzed in IBM SPSS Statistics 22 with a mixed-model ANOVA with the three within-subject factors: task (RET, MAN), load (L1, L3), and time window (1–4; Time); and the between-subject factor: group (NEG, POS, CON). This statistical procedure was repeated for each frequency band and each ROI separately. *Post-hoc* tests were Bonferroni corrected and when assumption of sphericity was not met data were Huynh-Feldt corrected.

#### Phase synchronization

In order to obtain theta phase values for calculations of the phase locking value (PLV) the phase of theta (center frequency of 6; 5 cycles complex continuous Morlet wavelet) was estimated between −pi and +pi in Brain Vision Analyzer 2.0 and then exported into Matlab to be further analyzed using in-house scripts. To assess whether theta phase estimated from the ACC is locked to the theta phases as obtained from the right and left BA40 the PLV was calculated according to Lachaux et al. (1999). The PLV is a measure of interregional phase synchronization by assessing the inter-trial variability of phase differences between signals from two distinct sources at any given time point. The PLV can range from zero to one, where a value of 1 indicates perfect stability of phase differences between the two signals across trials, and a value of 0 indicates completely random distribution of phase differences. First, the theta phase differences were calculated between two ROIs (frontal and right or left parietal) for every time point in every trial (2000 ms of delay period) for every condition and every participant separately. Then the values across trials were averaged and finally the data within the 2000 ms delay period were again averaged into four time windows of 500 ms each (see instantaneous amplitude). For statistical analysis the PLV values were analyzed in IBM SPSS Statistics 22 with a mixed-model ANOVA with the three within-subject factors: condition (RET, MAN), load (L1, L3), and time window (1–4); and the between-subject factor: group (NEG, POS, CON) for the right and the left BA40 separately.

## Results

### Behavioral results

The analysis of the behavioral data showed that the accuracy was significantly lower in the high-load (L3) than in the low-load (L1) condition in the retention task (*Mdn*_*L*1_ = 98.21, *Mdn*_*L*3_ = 97.32), *Z* = −2.38, *p* = 0.017, and in the manipulation task (*Mdn*_*L*1_ = 96.43, *Mdn*_*L*3_ = 83.04), *Z* = −4.79, *p* < 0.001. Further analysis showed that the accuracy was affected not only by the load, but also by the task. That is the accuracy was significantly lower in the manipulation task at low-load, *Z* = −2.71, *p* = 0.007 and at high load *Z* = −4.79, *p* < 0.001 in comparison to the retention task.

Importantly, the performed analysis on the accuracy between the three groups (negative symptomatic, positive symptomatic patients, and healthy controls) revealed no significant effect in either of the tasks at neither load. More specifically, the accuracy of the NEG, POS, and CON groups showed no significant difference in the retention task at low-load (L1), *H*_(2)_ = 3.28, *p* = 0.19, and at high-load (L3), *H*_(2)_ = 3.28, *p* = 0.19, or in the manipulation task at low-load (L1), *H*_(2)_ = 0.34, *p* = 0.846, and at high-load, *H*_(2)_ = 1.02, *p* = 0.602.

In sum the behavioral result suggest that accuracy was influenced by load and also by task. Participants performed better in the low-load conditions and in the retention task. Importantly, the three groups showed similar behavior performance, i.e., the control group performed no better than the patient groups (see Table 2 for accuracy values).

**Table 2:** **Mean accuracy with standard error across groups, conditions, and load**.

	**Retention**		**Manipulation**	
	***Load 1***	***Load 3***	***Load 1***	***Load 3***
CON	95.18 ± 3.25	93.04 ± 3.41	93.57 ± 2.58	81.79 ± 3.73
NEG	98.39 ± 0.62	97.50 ± 1.07	96.07 ± 0.95	83.22 ± 1.49
POS	97.14 ± 0.48	93.22 ± 2.01	94.64 ± 1.38	79.82 ± 2.61
Total	96.90 ± 1.10	94.58 ± 1.37	94.76 ± 1.01	81.61 ± 1.56

### Instantaneous amplitude

#### ACC theta

Analysis of the task-specific instantaneous theta amplitude values in the ACC revealed a main effect of Task, *F*_(1, 27)_ = 10.85, *p* = 0.003, indicating that the manipulation task was associated with higher theta amplitude in the ACC than the retention task. Furthermore, the main effect of Load was also significant, *F*_(1, 27)_ = 9.59, *p* = 0.005, because of the higher theta amplitude in the high-load (L3) condition. In addition, the also significant main effect of Time (1–4), *F*_(1.88, 50.7)_ = 10.94, *p* < 0.001, was the result of the attenuation of the theta amplitude increase (as to baseline) over the course of the delay period.

**Figure 2:**
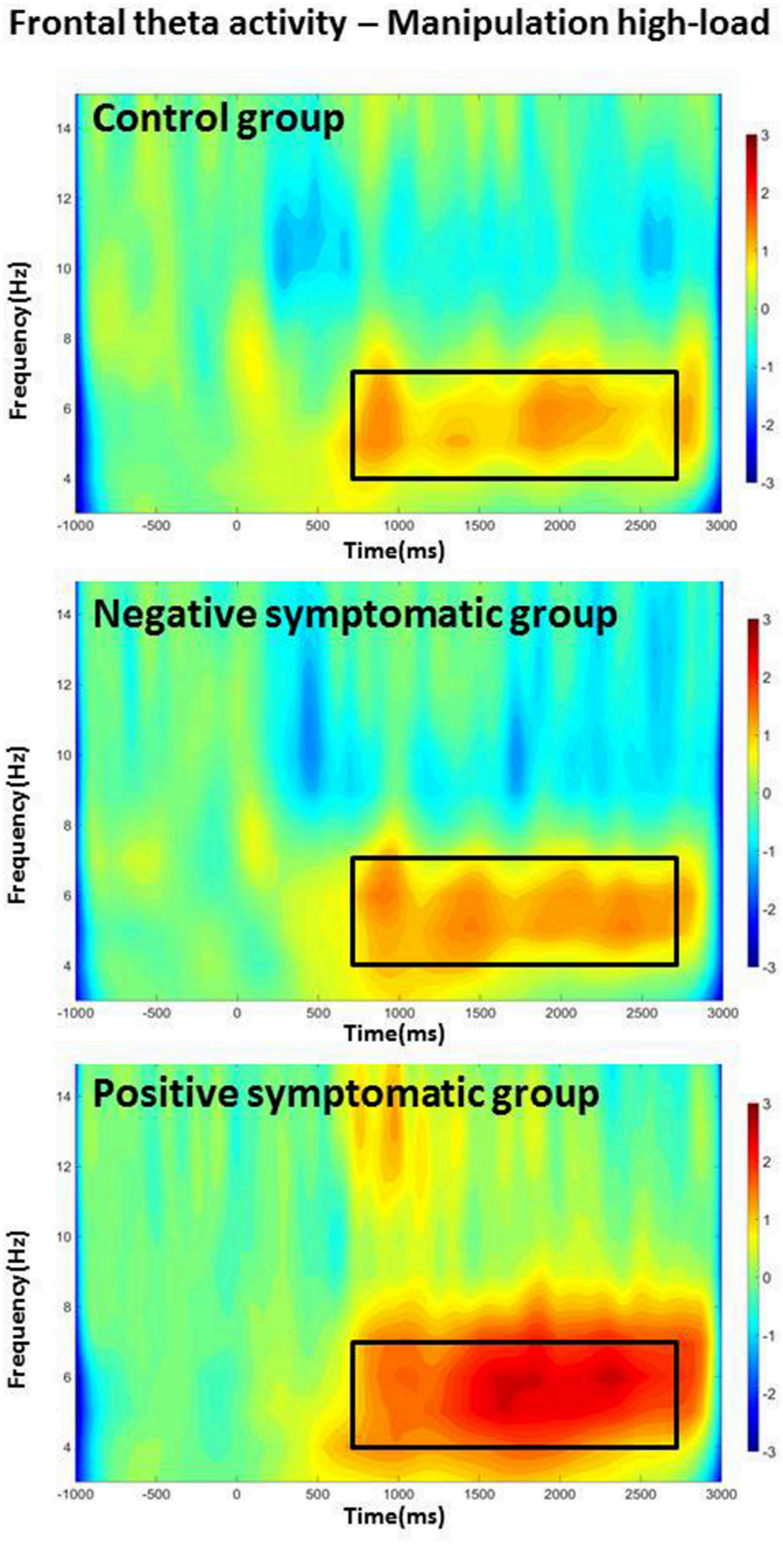
**Frontal theta band activity for the high-load manipulation condition for all three groups**. The frequency of interest (4–7 Hz) and time period of interest (delay period) are indicated with a frame.

In addition, interaction effects [Load × Time, *F*_(2.6, 70.18)_ = 10.69, *p* < 0.001; Task × Time interaction, *F*_(2.57, 68.74)_ = 8.2, *p* < 0.001] indicate that theta stays higher over the course of the whole delay period for the more difficult conditions (i.e., higher load and manipulation task). Finally, a Task × Load × Time × Group four-way interaction effect also reached significance, *F*_(5.95, 80.31)_ = 2.55, *p* = 0.026. Further testing showed that in the control group the theta power increase (as to baseline) showed consistent attenuation over the course of the delay period for the retention task in general and for the low-load condition of the manipulation task [main effect of Time *F*_RetL1(3, 27)_ = 8.87, *p* < 0.001; *F*_RetL3(3, 27)_ = 17.94, *p* < 0.001; *F*_ManL1(3, 27)_ = 13.41, *p* < 0.001]; whereas it did not drop over time in the high-load manipulation condition [Time *F*_ManL3(3, 27)_ = 0.9; *p* = 0.455]. The negative symptomatic group showed a pattern similar to the controls in the low-load retention condition [Time *F*_RetL1(1.3, 11.8)_ = 5.84, *p* = 0.026] but with a less pronounced attenuation of theta power. However, theta power was sustained not only in the manipulation high-load but also in the low-load and retention high-load condition [Time *F*_RetL3(1.8, 16.3)_ = 0.31, *p* = 0.715; *F*_ManL3(1.4, 12.9)_ = 0.1, *p* = 0.845; *F*_ManL1(3, 27)_ = 2.48, *p* = 0.083]. The positive symptomatic group displayed rather sustained theta power over the course of the delay period in all conditions except low-load retention condition [Time *F*_RetL1(3, 27)_ = 8.62, *p* < 0.001; *F*_RetL3(3, 27)_ = 2.22, *p* = 0.109; *F*_ManL1(3, 27)_ = 2.99, *p* = 0.052; *F*_ManL3(1.8, 16.6)_ = 1.29, *p* = 0.300]; with the high-load manipulation condition showing even a slight steady increase over the delay period (see Figure 2).

Overall, these results suggest that ACC theta amplitude was increased in the high-load condition and in the manipulation task and that theta amplitude was particularly pronounced during the early time windows of the delay period. Furthermore, the decrease over the course of the delay period was smaller in the high-load condition and in the manipulation task.

#### ACC gamma

The only effect that was significant in the analysis of the low-gamma band, was a main effect of Time, *F*_(2.56, 66.47)_ = 5.79, *p* = 0.002, indicating higher gamma power during the second part of the delay period, irrespective of task, load and group.

**Figure 3:**
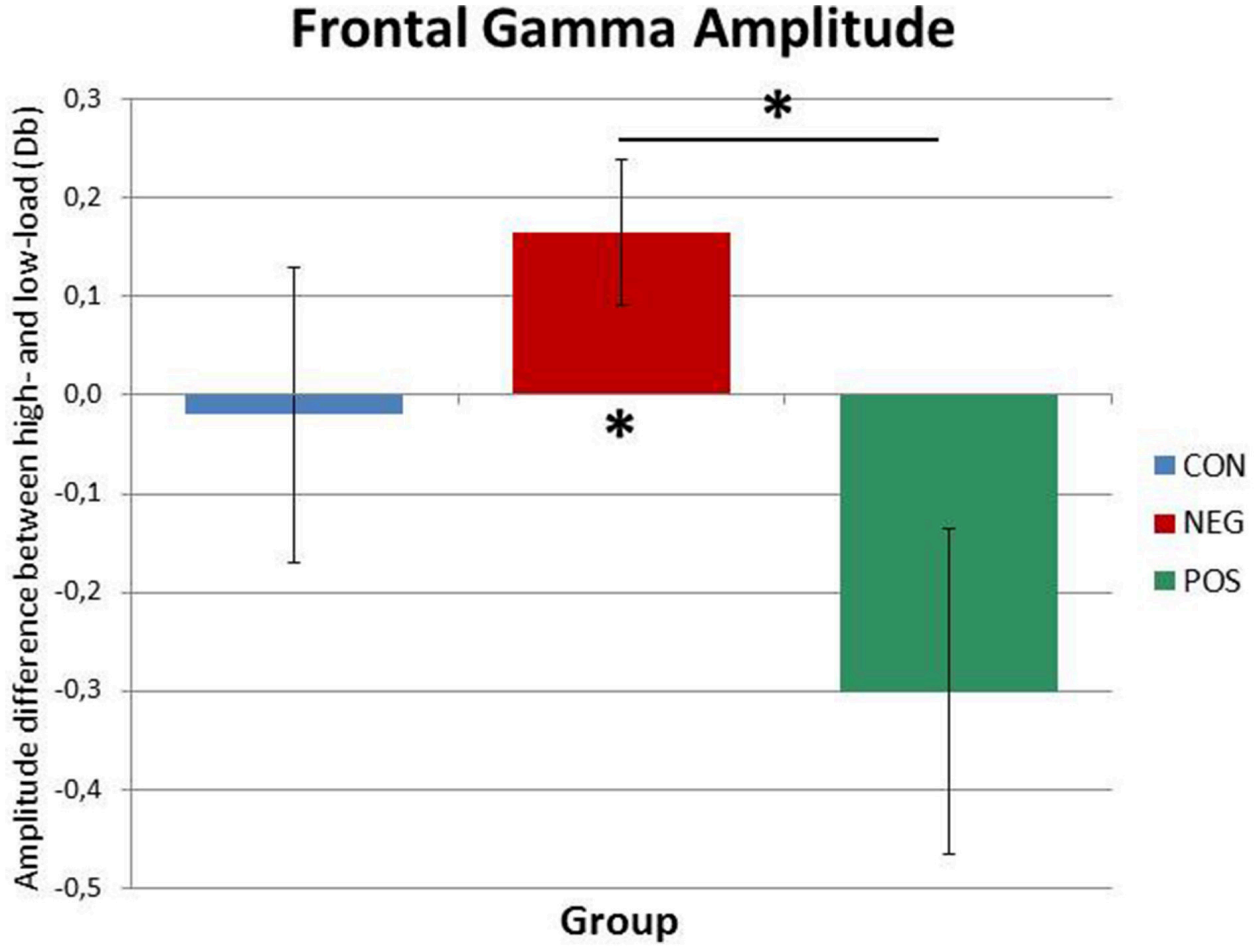
**Frontal gamma amplitude differences between high-load and low-load conditions for the three groups**. While the positive symptomatic group shows decreased gamma power in the ACC in the high-load condition, the negative symptomatic group displays a marked increase. The healthy control subjects do not differ from either patient group. Significant differences are indicated by asterisks (*p* < 0.05).

The analysis in the high gamma range revealed a main effect of Time, *F*_(1.92, 51.85)_ = 5.82, *p* = 0.006, which was driven by the increase of gamma power over the course of the delay period. Furthermore, central to our hypothesis, a marginally significant Load × Group interaction effect was found, *F*_(2, 27)_ = 3.1, *p* = 0.061 (see Figure 3). *Post-hoc* ANOVAs performed separately on low-load and high-load trials showed that whilst in the low-load condition, no significant main effect or interaction involving Group emerged, in the high-load condition a main effect of Group reached significance, *F*_(1, 27)_ = 3.48, *p* = 0.045. Importantly, the Bonferroni corrected (*p*_crit_ < 0.017) pairwise comparisons showed a significant difference only between positive and negative symptomatic groups, *t*_(18)_ = 2.65, *p* = 0.016, indicating higher gamma power in the negative symptomatic group than in the positive symptomatic group at high-load. In fact, further *post-hoc* repeated-measures ANOVA carried out on the positive and negative symptomatic group data separately suggested that, whereas in the negative symptomatic group the gamma power significantly increased from low-load to high-load conditions, *F*_(1, 9)_ = 6.29, *p* = 0.034, there was no difference in the positive symptomatic group gamma power across the same conditions (see Figure 3).

To summarize the above results, in the lower gamma frequency range the only significant finding suggested amplitude increase over the delay period. Similarly, high gamma amplitude also increased over the delay period; but importantly, amplitude was differentially affected by the factor group in the high- and low-load conditions. Whereas, in the low-load condition there was no difference among the groups, in the high-load condition gamma power was significantly higher in the negative symptomatic group than in the positive symptomatic group.

#### BA40 theta

Theta power in the right parietal ROI was modulated by several factors as a series of main and interaction effects showed. The strongly significant main effect of Time, *F*_(2.39, 64.62)_ = 11.57, *p* < 0.001, reflected the fact that theta amplitude was higher in the earlier time windows and showed a decrease over the course of the delay period.

Importantly, the results also indicated significant Time × Group, *F*_(4.79, 64.62)_ = 2.60, *p* = 0.035, and Task × Group interaction effects, *F*_(2, 27)_ = 3.55, *p* = 0.043. The former suggests that the decrease of theta power over the delay period was further modulated by the Group factor. Additional separate ANOVAs for each group individually revealed that there is no significant effect of Time in the healthy controls and the positive symptomatic group but a strongly significant main effect of Time in the negative symptomatic group *F*_(3, 9)_ = 13.73, *p* < 0.001; showing a decrease of theta power over the course of the delay period in the right BA40.

Furthermore, the above-mentioned significant Task × Group interaction effect was primarily driven by the fact that while the control group had increased theta power in the manipulation task compared to the retention task (irrespective of load), *F*_(1, 9)_ = 7.21, *p* = 0.025, the positive symptomatic group displayed a marginally significant decrease in the manipulation as compared to the retention task *F*_(1, 9)_ = 3.88, *p* = 0.08, and the negative symptomatic group displayed no significant difference.

Finally, the analysis of the left parietal ROI also showed a significant main effect of Time, *F*_(2.22, 59.87)_ = 31.65, *p* < 0.001 and a marginally significant main effect of Load, *F*_(1, 27)_ = 4.16, *p* = 0.051. The main effect of time indicated that over the course of the delay period theta power decreased.

To sum up, theta power in the right parietal ROI decreased over the delay period. This decrease was only significant in the negative symptomatic group but not in the other two groups. In addition, theta power was higher in the manipulation task compared to the retention task in the control group but not different between the two tasks in the patient groups.

#### BA40 gamma

No significant effects were found in the lower gamma band in the right BA40 and in the lower and high gamma band in the left BA40.

In the high gamma band the interaction effect Task × Load × Time was marginally significant *F*_(2.99, 80.76)_ = 2.7, *p* = 0.052 and the four-way interaction Task × Load × Time × Group reached a significant level *F*_(5.98, 80.76)_ = 3.99, *p* = 0.002 in the right BA40. Further investigation of the Task × Load × Time effect showed that while there was no task-dependent difference in right BA40 gamma power in the retention task (low- and high-load) and the high-load condition of the manipulation task; there was a marginally significant increase in the low-load condition of the manipulation task *F*_(2.2, 63.7)_ = 2.94, *p* = 0.055.

In order to explain the four-way interaction separate ANOVAs for the four conditions (RETL1, RETL3, MANL1, and MANL3) were run. No significant effects were found for the low-load and high-load retention and low-load manipulation conditions. In the high-load manipulation condition the controls displayed an increase in gamma amplitude over time, while the negative symptomatic group showed a decrease. The positive symptomatic group showed a pattern where there is a decrease in the early delay period and then an increase in the later time windows.

### Phase synchronization

The PLV of the theta frequency band between the ACC and right BA40 showed a highly significant Group effect, *F*_(1, 27)_ = 14.85, *p* < 0.001. Further *post-hoc* tests revealed that the control group had significantly higher PLV than the negative (*p* = 0.001), and also the positive symptomatic groups (*p* < 0.001), whereas the PLV did not differ significantly between the two patient groups (*p* = 0.654).

**Figure 4:**
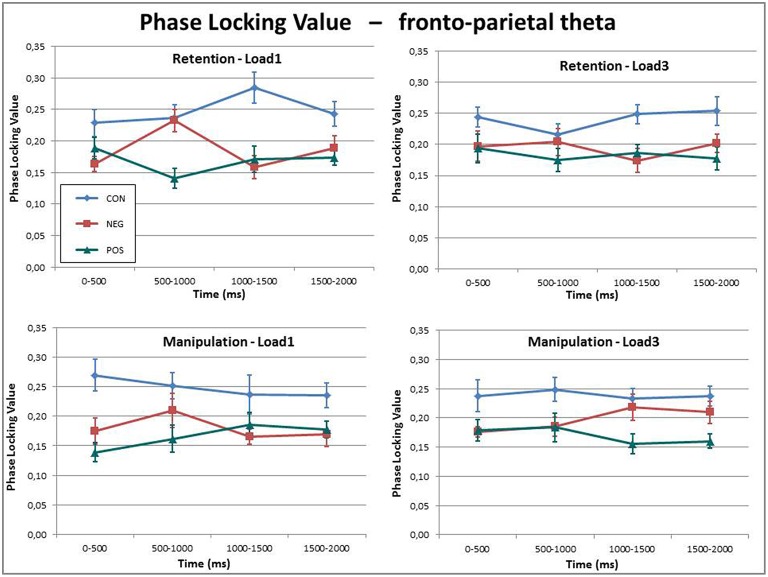
**Theta phase synchronization between the ACC and the BA40 indexed by phase locking values (PLVs) for all three groups in the four conditions over the delay period**. While the phase synchronization is fairly constant over time and between conditions, the healthy control subjects display a generally higher phase synchronization than the two patient groups.

In addition to this main effect, a significant Load × Time × Group, *F*_(6, 81)_ = 2.28, *p* = 0.044 three-way interaction effect was found (see Figure 4). Investigation of this three-way interaction indicated that the controls and positive symptomatic group displayed the same amount of theta phase synchronization over time for the low-load and high-load conditions while the negative symptomatic group showed a significant Load × Time interaction *F*_(3, 27)_ = 5.88, *p* = 0.003 indicating that there is a rather transient increase in phase synchronization in the low-load condition in the first half of the delay period. The high-load condition, on the other hand, shows a more sustained increase of phase synchronization over the course of the delay period.

Overall, these results indicate significantly stronger fronto-posterior theta phase synchronization in the healthy controls as compared to the patient groups.

## Discussion

Executive functions have previously been shown to employ a network of cortical structures spanning prefrontal areas, in particular the ACC, and right posterior parietal regions. This network is strongly involved in working memory and operations requiring cognitive control. Both, executive functions as well as the underlying neuronal network have been found to be compromised in schizophrenia. However, little is known about what it is exactly that underlies those dysfunctions. Furthermore, even less is known about the brain oscillatory correlates of the complex and highly diverse symptomatology of schizophrenia (i.e., negative and positive symptomatology) and its relation to executive functioning.

In this article we examined schizophrenia patients with predominantly negative symptomatology, with predominantly positive symptomatology and healthy control subjects. We recorded EEG while they performed a visuo-spatial delayed match to sample working memory task with varying load and varying need to employ executive functions. We investigated the delay period of the WM task and compared the three groups in respect to performance and brain oscillatory activity. We investigated areas of the fronto-parietal executive control network (ACC and right BA40 as well as left BA40) by looking at local amplitude changes of theta and gamma oscillations; two frequencies repeatedly found to be strongly implicated in visual working memory operations in these areas. Moreover, we investigated communication within this network by analysing phase synchrony in the theta frequency between the frontal and the parietal regions of interest. We hypothesized that patients would show a different pattern of functional cortical activation as compared to healthy controls. Furthermore, we expected the two patient groups to display differences in brain oscillatory correlates of executive functioning, given that their different symptomatologies greatly differ in their behavioral expression.

A very robust finding in this study is that sustained phase synchronization in the theta frequency between the ACC and the right (but not the left) BA40 was significantly disrupted in the patient groups as compared to controls (see Figure 4). Similar results were also obtained on scalp level by Griesmayr et al. (2014) where the 21 patients—as one sample—were compared with 21 matched healthy control subjects. Moreover, these findings are very well in line with the dysconnectivity theory of schizophrenia (e.g., Uhlhaas and Singer, 2010; Rogasch et al., 2014) stating that global cortical communication is disrupted in patients suffering from SZ. During working memory operations that require executive functions it is repeatedly found that theta oscillations in prefrontal areas are synchronized with theta oscillations in posterior parietal regions. This long-range communication is said to underlie (top–down) executive control from frontal areas interacting with local posterior information processing (e.g., Sauseng et al., 2005; Wu et al., 2007; Sauseng et al., 2008). Given that executive functions are often impaired in SZ, it stands to reason that such long-range theta coherence should also be disrupted. In our case, however, this pattern of dysconnectivity in SZ patients was not related to impaired task execution, i.e., patients did do just as well as the healthy control subjects.

In addition to theta long-range synchronization, the patients were also distinguishable from the healthy control subjects by the amount of local theta power difference between the retention and the manipulation tasks in the right BA40. While the healthy controls showed a markedly higher theta activity in the right posterior ROI in the manipulation task as compared to the retention task, the patient groups displayed no such load-dependent increase during the delay period. Posterior theta activity has been found to increase during working memory operations (e.g., Osipova et al., 2006; see Roux and Uhlhaas, 2014 for a review) and increasing load and has been linked to determining WM capacity (e.g., Moran et al., 2010). Hence, an increase in posterior theta activity from the retention to the manipulation task is not surprising. Such a task/load-dependent increase was also found in the ACC for all three groups; where theta power was significantly higher for the high-load and manipulation task as compared to low-load and retention task. What is curious, however, is that while patients displayed such task-dependent increase in theta power in the ACC, they did not show this pattern of increasing theta power in the BA40. Moreover, the positive symptomatic group was distinct from the healthy controls and the negative symptomatic group by the pattern of ACC theta activity mainly in the manipulation high-load condition (see Figure 2). The positive symptomatic patient group seemed to employ generally more sustained frontal theta activity in all conditions over time and even displayed an increase over the course of the delay period in the most difficult (high-load manipulation) condition. Given that frontal theta increase is a strong indicator for executive control (e.g., Gevins et al., 1997) this might hint at the employment of some sort of compensatory mechanism which recruits more executive processing power to successfully complete the task at hand.

Interestingly, we also found an effect in the high gamma frequency range in the ACC differentiating the two patient groups (see Figure 3). While gamma power did not differ between the three groups in the low-load conditions, the negative symptomatic patient group displayed significantly stronger gamma power than the positive symptomatic group in the high-load conditions. In fact, while local gamma power in the ACC increased from the low- to the high-load condition in the negative symptomatic group, the positive symptomatic group showed no difference between the two conditions. This finding suggests that local frontal information processing represented by gamma oscillatory activity seems to be aberrant in SZ and might be a key factor in differentiating the neural underpinnings of the different symptomatologies of the disorder. It is a finding well in line with the suggestion by Rogasch et al. (2014) that decreased prefrontal gamma activity is strongly linked to positive symptomatology. Furthermore, such differences between the patient groups might play a major role in explaining contradictory findings in literature where some authors find decreased frontal gamma in SZ while others find the opposite pattern (see Senkowski and Gallinat, 2015 for a review).

Another, although less robust effect indicated that theta power was attenuated over the time course of the delay period in the right parietal area in the negative symptomatic group whereas it stayed constant in the positive symptomatic group and in healthy control participants indicating consistent engagement with the to-be-retained information. As the area is thought to be strongly involved in visuospatial processing (Moscovitch et al., 1995) and there is evidence suggesting that the theta frequency band is particularly relevant in such processing (Romei et al., 2011), the disproportionate reduction of theta power could have a negative impact on visuospatial working memory. However, this different pattern in the negative symptomatic group does not necessarily reflect impairment: Firstly, their behavioral performance was not impaired as compared to the other two groups; and secondly, they seem to show a generally slightly different oscillatory pattern in this task than controls and positive symptomatic patients. This might partly reflect parallel mechanisms to compensate for the comprised parietal theta activity (see for instance the ACC gamma activity differences between negative symptomatic and the other two groups). To draw any clear conclusions further studies would be needed to investigate the exact nature and relevance of this effect more in depth.

In the current study we only found local and fronto-parietal coupling results in the right but not the left parietal ROI. This might be because of the material type (i.e., strictly spatial information processing) or the required attention network. The right parietal region is strongly linked to visuospatial processing and moreover to sustained attention to spatial locations (e.g., Smith et al., 1996; Malhotra et al., 2009; Jackson et al., 2011). A right hemispheric dominance for visuospatial processing, representation and working memory has been shown in both, primates and humans with neuropsychological and neuroimaging techniques as well as in neurostimulation studies (e.g., Pisella et al., 2011; for reviews see Oleksiak et al., 2011; Pisella, 2016). However, we want to point out that the role of the right parietal cortex in visuospatial cognition is not yet fully understood and that visual working memory processing and attention is not exclusively right lateralised (e.g., Cowan et al., 2011 for color processing; Majerus et al., 2010 for faces; Popov et al., 2015 for executive functioning).

Generally, our results suggest that patients suffering from schizophrenia display altered oscillatory patterns during executive processes indicated by disrupted fronto-parietal theta synchronization in the executive network and local right parietal theta activity, indicating differential local neural processes. Importantly, local ACC gamma and to a lesser extent right parietal and ACC theta also have differential patterns between positive and negative symptomatic SZ patients, but long-range fronto-parietal coupling did not distinguish the two patient groups but only patients and healthy controls. It is important to point out that our data mainly identified neuronal processes inherent to patients suffering from predominantly negative symptomatology which was the group the most distinguishable from both, the healthy controls and the positive symptomatic group in local neuronal processing (see group effects in ACC gamma and right BA40 theta). That this group stands out is not surprising given that the two patient groups only differ significantly in the negative symptoms (see Table 1) while their positive symptomatology scores are not significantly different. The fact that the performance between groups was not different points toward the aberrant neuronal processing displayed by the negative symptomatic group really being partly of a compensatory nature; however, from our data we cannot make any definite conclusions about the meaning of different oscillatory patterns or suggestions regarding causality. Given that aberrant gamma oscillations are linked to a dysregulation of GABAergic inhibitory neurotransmission, the differential findings in the gamma frequency band between the two patient groups might have potential implications for medication treatment of the SZ subgroups in the future.

We argue that the set of results might have very distinct origins and functions. The pattern of dysconnectivity in SZ which we also found in this study might be explained by general white matter loss in patients (e.g., Roalf et al., 2015), irrespective of symptomatology. However, diffusion tensor imaging studies would be needed to confirm such a claim. With regards to the local effects we found in right parietal and ACC theta power and frontal gamma power, this might reflect either on local disruptions (in the case of parietal theta activity) or alternatively, compensatory mechanisms in order to counter processing deficits. The increased frontal gamma and theta activity in the negative symptomatic group and positive symptomatic group, respectively, fits nicely with findings suggesting an increase in frontal activity as compensatory mechanism (i.e., hyperfrontality) as gamma activity has been found to reflect local neuronal spiking and frontal theta activity is strongly linked to executive functions.

It is important to emphasize that the above findings are unlikely to represent only dysfunctional neural processes. Disruption of a neural network may also trigger compensatory mechanisms. Thus, in particular the here described local differences between SZ patients and healthy controls, as well as, between the negative and positive symptomatic patient groups can represent local disruption or local compensation mechanisms. Considering that the negative and positive patient groups' behavioral performance was at the level of the healthy controls, the presence of such compensatory mechanisms is more than likely. However, it is also important to note that in the present study the healthy control sample was a tight match to the patient sample. While most studies comparing SZ patients and healthy controls report that patients perform markedly worse than healthy controls, the non-existent group difference in our study might indicate that no other factors (e.g., general cognitive abilities) contributed to the differences in brain oscillatory activity we found.

Furthermore, it is relevant to point out that the negative and positive patient groups received various psychopharmacological medications, which could potentially influence oscillatory processes; especially as there was a significant difference between the two patient groups. Hence it cannot be excluded that our results may be affected by these medications. However, the impact of medications on oscillatory processes is most likely generic and not task-specific. The presented results were specific to executive functions in a working memory task and do not reflect on generally altered oscillatory patterns as investigated in studies looking at resting EEG activity. It might also be worth mentioning that during the baseline period in our task there was no significant difference in either frequency band between groups. Moreover, it was found that prefrontal theta and gamma changes are not associated with psychopharmacological treatment in first-episode SZ patients (e.g., Minzenberg et al., 2010) and that deficits are not just due to lack of concentration, distracting positive symptoms or medication effects (e.g., Kraguljac et al., 2013).

Finally, we want to highlight that our analysis was strictly hypothesis driven. That is we did only analyse regions and frequency bands that are strongly implicated in fronto-parietal executive functions and visual working memory operations that are easily accessible with EEG source localization; i.e., the ACC and BA40. We cannot exclude, however, that other regions might play an important role and show distinct patterns in patients and controls, and more to the point, between the two patient groups. This would need to be further investigated with a more exploratory approach and potentially wider range of cognitive tasks and neuroimaging methods. In addition, in order to be able to make definite claims our results would need to be investigated further with a much larger sample of patients and healthy controls.

## Conclusion

In conclusion, our results indicate that indeed, the fronto-parietal executive functions network as indexed by theta phase synchronization is comprised in SZ. Moreover, local frontal and right parietal activity in the theta and gamma frequency ranges can distinguish between patients with predominantly negative and predominantly positive symptomatology; and are especially able to differentiate patients with negative symptomatology from patients with positive symptoms and healthy controls. Finally, task-related theta and gamma oscillations can be highly beneficial for understanding the multifaceted nature of SZ and how its various cognitive dysfunctions and symptoms emerge—even in the absence of behavioral impairment—and so in furthering its treatment.

## Author contributions

BB and TM share the first authorship for this paper and contributed equally. BB, data collection, data analysis, writing manuscript; TM, data analysis, writing manuscript; BG, implementation of experiment, data collection, writing manuscript; RS, WA, implementation of experiment, writing manuscript; PS, implementation of experiment, data analysis, writing manuscript.

## Funding

This research was supported by the DFG (SA1872/2-1) for PS and BG was recipient of a Doc fFORTE fellowship by the Austrian Academy of Sciences.

### Conflict of interest statement

The authors declare that the research was conducted in the absence of any commercial or financial relationships that could be construed as a potential conflict of interest.

## References

[B1] Basar-Eroglu C. Brand A. Hildebrandt H. Kedzior K. K. Mathes B. Schmiedt C. (2007). Working memory related gamma oscillations in schizophrenia patients. Int. J. Psychophysiol. 64, 39–45. 10.1016/j.ijpsycho.2006.07.007 16962192

[B2] Berger B. Omer S. Minarik T. Sterr A. Sauseng P. (2014). Interacting memory systems – does EEG alpha activity respond to semantic long-term memory access in a working memory task? Biology 4, 1–16. 10.3390/biology4010001 25545793 PMC4381213

[B3] Buzsaki G. Wang X.-J. (2012). Mechanisms of gamma oscillations. Annu. Rev. Neurosci. 35, 203–225. 10.1146/annurev-neuro-062111-150444 22443509 PMC4049541

[B4] Callicott J. H. Mattay V. S. Verchinski B. A. Marenco S. Egan M. F. Weinberger D. R. (2003). Complexity of prefrontal cortical dysfunction in schizophrenia: More than up or down. Am. J. Psychiatry 160, 2209–2215. 10.1176/appi.ajp.160.12.2209 14638592

[B5] Carter C. S. Mintun M. Nichols T. Cohen J. D. (1997). Anterior cingulate gyrus dysfunction and selectrive attentino deficit in schizophrenia: [15 O]H2O PET study during single-trial stroop task performance. Am. J. Psychiatry 154, 1670–1675. 10.1176/ajp.154.12.1670 9396944

[B6] Cavanagh J. G. Frank M. J. (2014). Frontal theta as a mechanism for cognitive control. Trends Cogn. Sci. 18, 414–421. 10.1016/j.tics.2014.04.012 24835663 PMC4112145

[B7] Cho R. Y. Konecky R. O. Carter C. S. (2006). Impairments in frontal cortical γ synchrony and cognitive control in schizophrenia. Proc. Natl. Acad. Sci. U.S.A. 103, 19878–19883. 10.1073/pnas.0609440103 17170134 PMC1750867

[B8] Cohen M. X. Ridderinkhof K. R. (2013). EEG source reconstruction reveals frontal-parietal dynamics of spatial conflict processing. PLoS ONE 8:e57293. 10.1371/journal.pone.0057293 23451201 PMC3581478

[B9] Cowan N. Li D. Moffitt A. Becker T. M. Martin E. A. Saults J. S.. (2011). A neural region of abstract working memory. J. Cogn. Neurosci. 23, 2852–2863. 10.1162/jocn.2011.21625 21261453 PMC3138911

[B10] Dasklakis Z. J. Fitzgerald P. B. Christensen B. K. (2007). The role of cortical inhibition in the pathophysiology and treatment of schizophrenia. Brain Res. Rev. 56, 427–442. 10.1016/j.brainresrev.2007.09.006 17980435

[B11] Etkin A. Gyurak A. O'Hara R. (2013). A neurobiological approach to the cognitive deficits of psychiatric disorders. Dialogues Clin. Neurosci. 15, 419–429. 24459409 10.31887/DCNS.2013.15.4/aetkin PMC3898680

[B12] Ettinger U. Mohr C. Gooding D. C. Cohen A. S. Rapp A. Haenschel C.. (2015). Cognition and brain function in schizotypy: a selective review. Schizophr. Bull. 41, 417–426. 10.1093/schbul/sbu190 25810056 PMC4373634

[B13] Franke G. H. (2000). BSI. Brief Symptom Inventory von L. R. Derogatis Deutsche Version. Göttingen: Beltz.

[B14] Gandal M. J. Edgar J. C. Klook K. Siegel S. J. (2012). Gamma synchrony: towards a translational biomarker for the treatment resistant symptoms of schizophrenia. Neuropharmacology 62, 1504–1518. 10.1016/j.neuropharm.2011.02.007 21349276 PMC3264822

[B15] Gevins A. Smith M. E. McEvoy L. Yu D. (1997). High-resolution EEG mapping of cortical activation related to working memory: effects of task difficulty, type of processing, and practice. Cereb. Cortex 7, 374–385. 10.1093/cercor/7.4.374 9177767

[B16] Glahn D. D. Ragland J. D. Abramoff A. Barrett J. Laird A. R. Bearden C. E.. (2005). Beyond hypofrontality: a quantitative meta-analysis of functional neuroimaging studies of working memory in schizophrenia. Hum. Brain Mapp. 25, 60–69. 10.1002/hbm.20138 15846819 PMC6871703

[B17] Griesmayr B. Berger B. Stelzig-Schoeler R. Aichhorn W. Bergmann J. Sauseng P. (2014). EEG theta phase coupling during executive control of visual working memory investigated in individuals with schizophrenia and in healthy controls. Cogn. Affect. Behav. Neurosci. 14, 1340–1355. 10.3758/s13415-014-0272-0 24763921

[B18] Haenschel C. Bittner R. A. Waltz J. Haertling F. Wibral M. Singer W.. (2009). Cortical oscillatory activity is critical for working memory as revealed by deficits in early-onset schizophrenia. J. Neurosci. 29, 9481–9489. 10.1523/JNEUROSCI.1428-09.2009 19641111 PMC6666530

[B19] Haenschel C. Linden D. (2011). Exploring intermediate phenotypes with EEG: working memory dysfunction in schizophrenia. Behav. Brain Res. 216, 481–495. 10.1016/j.bbr.2010.08.045 20816898

[B20] Herrmann C. S. Demiralp T. (2005). Human EEG gamma oscillations in neuropsychiatric disorders. Clin. Neurophysiol. 116, 2719–2733. 10.1016/j.clinph.2005.07.007 16253555

[B21] Huhlshoff Pol H. E. Schnack H. G. Bertens M. G. van Haren N. E. van der Tweel I. Staal W. G.. (2002). Volume changes in gray matter in patients with schizophrenia. Am. J. Psychiatry 159, 244–250. 10.1176/appi.ajp.159.2.244 11823266

[B22] Ishii R. Canuet L. Ishihara T. Aoki Y. Ikeda S. Hata M.. (2014). Frontal midline theta rhythm and gamma power changes during focused attention on mental calculation: an MEG beamformer analysis. Front. Hum. Neurosci. 8:406. 10.3389/fnhum.2014.00406 24966825 PMC4052629

[B23] Jackson M. C. Morgan H. M. Shapiro K. L. Mohr H. Linden D. E. J. (2011). Strategic resource allocation in the human brain supports cognitive coordination of object and spatial working memory. Hum. Brain Mapp. 32, 1330–1348. 10.1002/hbm.21112 20715083 PMC3326378

[B24] Karlsgodt K. H. Glahn D. C. van Erp T. G. M. Therman S. Huttunen M. Manninen M.. (2007). The relationship between performance and fMRI signal during working memory in patients with schizophrenia, unaffected co-twins, and control subjects. Schizophr. Res. 89, 191–197. 10.1016/j.schres.2006.08.016 17029749

[B25] Kay S. R. Fiszbein A. Opler L. A. (1987). The positive and negative syndrome scale (PANSS) for schizophrenia. Schizophr. Bull. 13, 261–276. 10.1093/schbul/13.2.261 3616518

[B26] Kerns J. G. Cohen J. D. MacDonald A. W. III Johnson M. K. Stenger V. A. Aizenstein H.. (2005). Decreased conflict- and error-related activity in the anterior cingulate cortex in subjects with schizophrenia. Am. J. Psychiatry 162, 1833–1839. 10.1176/appi.ajp.162.10.1833 16199829

[B27] Kiriakopoulos M. Bargiotas T. Barker G. J. Frangou S. (2008). Diffusion tensor imaging in schizophrenia. Eur. Psychiatry 23, 255–273. 10.1016/j.eurpsy.2007.12.004 18524546

[B28] Koychev I. El-Deredy W. Mukherjee T. Haenschel C. Deakin J. F. W. (2012). Core dysfunction in schizophrenia: electrophysiology trait biomarkers. Acta Psychiatr. Scand. 126, 59–71. 10.1111/j.1600-0447.2012.01849.x 22384856

[B29] Kraguljac N. V. Srivastava A. Lahti A. C. (2013). Memory deficits in schizophrenia: a selective review of functional magnetic resonance imaging (FMRI) studies. Behav. Sci. (Basel) 3, 330–347. 10.3390/bs3030330 25379242 PMC4217593

[B30] Lachaux J.-P. Rodriguez E. Martinerie J. Varela F. J. (1999). Measuring phase synchrony in brain signals. Hum. Brain Mapp. 8, 194–208. 10619414 10.1002/(SICI)1097-0193(1999)8:4<194::AID-HBM4>3.0.CO;2-C PMC6873296

[B31] Lisman J. (2012). Excitation, inhibition, local oscillations, or large-scale loops: what causes the symptoms of schizophrenia? Curr. Opin. Neurobiol. 22, 537–544. 10.1016/j.conb.2011.10.018 22079494 PMC3302967

[B32] Lisman J. Buzsaki G. (2008). A neural coding scheme formed by the combined function of gamma and theta oscillations. Schizophr. Bull. 34, 974–980. 10.1093/schbul/sbn060 18559405 PMC2518638

[B33] Majerus S. D'Argembeau A. Perez T. M. Belayachi S. van der Linden M. Collette F.. (2010). The commonality of neural networks of verbal and visual short-term memory. J. Cogn. Neurosci. 22, 2570–2593. 10.1162/jocn.2009.21378 19925207

[B34] Malhotra P. Coulthard E. J. Husain M. (2009). Role of right posterior parietal cortex in maintaining attention to spatial locations over time. Brain 132, 645–660. 10.1093/brain/awn350 19158107 PMC2664449

[B35] Manoach D. S. (2003). Prefrontal cortex dysfunction during working memory performance in schizophrenia: reconciling discrepant findings. Schizophr. Res. 60, 285–298. 10.1016/S0920-9964(02)00294-3 12591590

[B36] Manoach D. S. Gollub R. L. Benson E. S. Searl M. M. Goff D. C. Halpern E.. (2000). Schizophrenic subjects show aberrant fMRI activation of dorsolateral prefrontal cortex and basal ganglia during working memory performance. Biol. Psychiatry 48, 99–109. 10.1016/S0006-3223(00)00227-4 10903406

[B37] Meyer-Lindenberg A. Poline J. B. Kohn P. D. Holt J. L. Egan M. F. Weinberger D. R.. (2001). Evidence for abnormal cortical functional connectivity during working memory in schizophrenia. Am. J. Psychiatry 158, 1809–1817. 10.1176/appi.ajp.158.11.1809 11691686

[B38] Minzenberg M. J. Firl A. J. Yoon J. H. Gomes G. C. Reinking C. Carter C. S. (2010). Gamma oscillatory power is impaired during cognitive control independent of medication status in first-episode schizophrenia. Neuropsychopharmacology 35, 2590–2599. 10.1038/npp.2010.150 20827271 PMC3055567

[B39] Mitchell D. J. McNaughton N. Flanagan D. Kirk I. J. (2008). Frontal-midline theta from the perspective of hippocampal “theta”. Prog. Neurobiol. 86, 156–185. 10.1016/j.pneurobio.2008.09.005 18824212

[B40] Mizuhara H. Yamagutchi Y. (2007). Human cortical circuits for central executive function emerge by theta phase synchronization. Neuroimage 36, 232–244. 10.1016/j.neuroimage.2007.02.026 17433880

[B41] Möller H.-J. Müller W. E. Volz H.-P. (2000). Psychopharmakotherapie. Ein Leitfaden für Klinik und Praxis. Stuttgart: Kohlhammer.

[B42] Moran L. V. Hong L. E. (2011). High vs low frequency neural oscillations in schizophrenia. Schizophr. Bull. 37, 659–663. 10.1093/schbul/sbr056 21653278 PMC3122299

[B43] Moran R. J. Campo P. Maestu F. Reilly R. B. Dolan R. J. Strange B. A. (2010). Peak frequency in the theta and alpha bands correlates with human working memory capacity. Front. Hum. Neurosci. 4:200. 10.3389/fnhum.2010.00200 21206531 PMC3009479

[B44] Moscovitch M. Kapur S. Köhler S. Houle S. (1995). Distinct neural correlates of visual long-term memory for spatial location and object identity: a positron emission tomography study in humans. Proc. Natl. Acad. Sci. U.S.A. 92, 3721–3725. 10.1073/pnas.92.9.3721 7731972 PMC42033

[B45] O'Keefe J. Recce M. L. (1993). Phase relationship between hippocampal place units and the EEG theta rhythm. Hippocampus 3, 317–330. 10.1002/hipo.450030307 8353611

[B46] Oleksiak A. Postma A. van der Ham I. J. Klink P. C. van Wezel R. J. (2011). A review of lateralization of spatial functioning in nonhuman primates. Brain Res. Rev. 67, 56–72. 10.1016/j.brainresrev.2010.11.002 21059373

[B47] Osipova D. Takashima A. Oostenveld R. Fernández G. Maris E. Jensen O. (2006). Theta and gamma oscillations predict encoding and retrieval of declarative memory. J. Neurosci. 26, 7523–7531. 10.1523/JNEUROSCI.1948-06.2006 16837600 PMC6674196

[B48] Pascual-Marqui R. D. Michel C. M. Lehmann D. (1994). Low resolution electromagnetic tomography: a new method for localizing electrical activity in the brain. Int. J. Psychophysiol. 18, 49–65. 10.1016/0167-8760(84)90014-X 7876038

[B49] Pisella L. (2016). Visual perception is dependent on visuospatial working memory and thus on the posterior parietal cortex. Ann. Phys. Rehabil. Med. [Epub ahead of print]. 10.1016/j.rehab.2016.01.002 26926263

[B50] Pisella L. Alahyane N. Blangero A. Thery F. Blanc S. Pelisson D. (2011). Right-hemispheric dominance for visual remapping in humans. Philos. Trans. R. Soc. Lond. B. Biol. Sci. 366, 572–585. 10.1098/rstb.2010.0258 21242144 PMC3030835

[B51] Popov T. Wienbruch C. Meissner S. Miller G. A. Rockstroh B. (2015). A mechanism of deficient interregional neural communication in schizophrenia. Psychophysiology 52, 648–656. 10.1111/psyp.12393 25495241

[B52] Roalf D. R. Gur R. E. Verma R. Parker W. W. Quarmley M. Ruparel K. (2015). White matter microstructure in schizophrenia: Associations to neurocognitive and clinical symptomatology. Schizophr. Res. 161, 42–49. 10.1016/j.schres.2014.09.026 25445621 PMC4410368

[B53] Rogasch N. C. Daskalakis Z. J. Fitzgerald P. B. (2014). Cortical inhibition, excitation, and connectivity in schizophrenia: a review of insights from transcranial magnetic stimulation. Schizophr. Bull. 40, 685–696. 10.1093/schbul/sbt078 23722199 PMC3984517

[B54] Romei V. Driver J. Schyns P. G. Thut G. (2011). Rhythmic TMS over right parietal cortex causally links distinct brain frequencies to global visual processing. Curr. Biol. 21, 334–337. 10.1016/j.cub.2011.01.035 21315592 PMC3063337

[B55] Roux F. Uhlhaas P. J. (2014). Working memory and neural oscillations: alpha-gamma versus theta-gamma codes for distinct WM information? Trends Cogn. Sci. 18, 16–25. 10.1016/j.tics.2013.10.010 24268290

[B56] Sauseng P. Griesmayr B. Freunberrger R. Klimesch W. (2010). Control mechansims in working memory: A possible function of EEG theta oscillations. Neurosci. Biobehav. Rev. 34, 1015–1022. 10.1016/j.neubiorev.2009.12.006 20006645

[B57] Sauseng P. Hoppe J. Klimesch W. Gerloff C. Hummel F. C. (2007). Dissociation of sustained attention from central executive functions: local activity and interregional connectivity in the theta range. Eur. J. Neurosci. 25, 587–593. 10.1111/j.1460-9568.2006.05286.x 17284201

[B58] Sauseng P. Klimesch W. Gruber W. Birbaumer N. (2008). Cross-frequency phase synchronization: a brain mechanism of memory matching and attention. Neuroimage 40, 308–317. 10.1016/j.neuroimage.2007.11.032 18178105

[B59] Sauseng P. Klimesch W. Schabus M. Doppelmayr M. (2005). Fronto-parietal EEG coherence in theta and upper alpha reflect central executive functions of working memory. Int. J. Psychophysiol. 57, 97–703. 10.1016/j.ijpsycho.2005.03.018 15967528

[B60] Schmitt A. Hasan A. Gruber O. Falkai P. (2011). Schizophrenia is a disorder or disconnectivity. Eur. Arch. Psychiatry 261, s150–s154. 10.1007/s00406-011-0242-2 21866371 PMC3207137

[B61] Senkowski D. Gallinat J. (2015). Dysfunctional prefrontal gamma-band oscillations reflect working memory and other cognitive deficits in schizophrenia. Biol. Psychiatry 77, 1010–1019. 10.1016/j.biopsych.2015.02.034 25847179

[B62] Singer W. Gray C. M. (1995). Visual feature integration and the temporal correlation hypothesis. Annu. Rev. Neurosci. 18, 555–586. 10.1146/annurev.ne.18.030195.003011 7605074

[B63] Smith E. E. Jonides J. Koeppe R. A. (1996). Dissociating verbal and spatial working memory using PET. Cereb. Cortex 6, 11–20. 10.1093/cercor/6.1.11 8670634

[B64] Teffer K. Semendeferi K. (2012). Human prefrontal cortex: evolution, development, and pathology. Prog. Brain Res. 195, 191–218. 10.1016/B978-0-444-53860-4.00009-X 22230628

[B65] Tsujimoto T. Shimazu H. Isomura Y. (2006). Direct recording of theta oscillations in primate prefrontal and anterior cingulate cortices. J. Neurophysiol. 95, 2987–3000. 10.1152/jn.00730.2005 16467430

[B66] Uhlhaas P. J. Singer W. (2010). Abnormal neural oscillations and synchrony in schizophrenia. Nat. Rev. Neurosci. 11, 100–113. 10.1038/nrn2774 20087360

[B67] Uhlhaas P. J. Singer W. (2014). Oscillations and neuronal dynamics in schizophrenia: the search for basic symptoms and translational opportunities. Biol. Psychiatry 77, 1001–1009. 10.1016/j.biopsych.2014.11.019 25676489

[B68] Uhlhaas P. J. Singer W. (2015). Oscillations and neuronal dynamics in schizophrenia: the search for basic symptoms and translational opportunities. Biol. Psychiatry 77, 1001–1009. 10.1016/j.biopsych.2014.11.019 25676489

[B69] van Erp T. G. M. Hibar D. P. Rasmussen J. M. Glahn D. C. Pearlson D. Andreassen O. A. (2015). Subcortical brain volume abnormalities in 2028 individuals with schizophrenia and 2540 healthy controls via the ENIGMA consortium. Mol. Psychiatry 2015, 1–7. 10.1038/mp.2015.63 PMC4668237 26033243

[B70] Voytek B. Knight R. T. (2015). Dynamic network communication as a unifying neural basis for cognition, development, aging, and disease. Biol. Psychiatry 77, 1089–1097. 10.1016/j.biopsych,.2015.04.016 26005114 PMC4443259

[B71] Wang L. Liu X. Guise K. G. Knight R. Ghajar J. Fan J. (2009). Effective connectivity of the fronto-parietal network during attention control. J. Cogn. Neurosci. 22, 543–553. 10.1162/jocn.2009.21210 19301995

[B72] Wen W. Yong H. Sachdev P. (2011). Structural brain networks and neuropsychiatric disorders. Curr. Opin. Psychiatry 24, 219–225. 10.1097/YCO.0b013e32834591f8 21430538

[B73] Woods S. W. (2003). Chlorpromazine equivalent doses for the newer atypical antipsychotics. J. Clin. Psychiatry 64, 663–667. 10.4088/JCP.v64n0607 12823080

[B74] Wu X. Chen X. Li Z. Han S. Zhang D. (2007). Binding of verbal and spatial information in human working memory involves large-scale neural synchronization at theta frequency. Neuroimage 35, 1654–1662. 10.1016/j.neuroimage.2007.02.011 17379539

[B75] Zhou Y. Fan L. Qiu C. Jiang T. (2015). Prefrontal cortex and the dysconnectivity hypothesis of schizophrenia. Neurosci. Bull. 31, 207–219. 10.1007/s12264-014-1502-8 25761914 PMC5563697

